# Independent Motion Segmentation Based on Pure Event Data

**DOI:** 10.3390/s26092620

**Published:** 2026-04-23

**Authors:** Wenjun Yin, Dongdong Teng, Lilin Liu

**Affiliations:** 1 State Key Lab of Optoelectronic Materials and Technology, School of Electronics and Information Technology, Sun Yat-Sen University, Xin’gang West Road No. 135, Guangzhou 510275, China; yinwj7@mail2.sysu.edu.cn; 2 School of Physics, Sun Yat-Sen University, Xin’gang West Road No. 135, Guangzhou 510275, China

**Keywords:** event camera, independent motion segmentation, principal component analysis, event leaky integration model

## Abstract

Event cameras are bio-inspired vision sensors offering low latency, low power consumption, and high dynamic range, capturing motion with microsecond-level precision via a per-event triggering mechanism. Despite these advantages, the inherent sparsity and lack of color in event data hinder direct analysis, necessitating advanced deep learning approaches. To achieve low-latency and high-precision motion segmentation for indoor robotic applications, this paper introduces a dual-branch decoupled CNN framework. Specifically, Principal Component Analysis (PCA) is utilized to project 3D event point clouds into 2D motion trend maps, capturing local motion priors while suppressing ambiguity in structured environments. Concurrently, an Event Leaky Integration (ELI) model, inspired by biological membrane potentials, is designed to enhance the structural representation of sparse events. Within this framework, separate branches respectively perform motion validation and shape extraction and are fused via a Spatial Gated Fusion (SGF) module to suppress static background interference. It is demonstrated experimentally that with an input window of only 10 ms, the proposed method achieves a 77% average mIoU across five indoor test scenarios from the EV-IMO dataset with an inference latency of 10 ms per frame. Compared to state-of-the-art methods like MSRNN and GCN, which required 30–300 ms event slices, our framework achieves a favorable trade-off between computational efficiency and segmentation accuracy, maintaining competitive performance under ultra-short time windows for indoor event-based motion processing.

## 1. Introduction

### 1.1. Biological Inspiration for Motion Perception

In the evolutionary trajectory of biological visual systems, dynamic motion perception has emerged as a critical survival capability. Athletes, for instance, precisely estimate projectile trajectories during high-speed locomotion, while raptors and insects rapidly track prey or evade predators. The essence of this capability lies in specialized functional regions that compute and neutralize ego-motion-induced background interference—a process known as ego-motion compensation. This mechanism allows biological systems to isolate the true motion of external objects, offering profound bio-inspired insights for enhancing the perceptual robustness of robotics, UAVs, and autonomous vehicles in complex environments [1,2].

### 1.2. Limitations of Conventional Sensors

Traditional motion segmentation relies primarily on RGB cameras and LiDAR, focusing on real-time performance, blur resistance, and adaptability to sparse scenes [3]. However, RGB-based methods are constrained by fixed frame rates (typically 24–60 fps), leading to motion blur and segmentation latency during rapid movements. Although high-speed cameras (up to 1000 Hz) exist, they introduce excessive data redundancy and computational overhead. Furthermore, implementing adaptive frame rates remains difficult for frame-based sensors due to the challenges of real-time trigger determination. LiDAR-based methods often require prior semantic segmentation and struggle with non-rigid motions (e.g., limb movements or vehicle turns) because point clouds frequently lack sufficient temporal continuity [4].

### 1.3. Challenges in Event-Based Vision

Event cameras, as bio-inspired sensors, transcend the traditional frame-based paradigm. Their microsecond-level temporal resolution captures instantaneous dynamics, mitigating latency, while their asynchronous nature—generating data only where intensity changes—suppresses static noise and reduces computational costs [5,6].

Despite these advantages, current event-based motion segmentation, which largely relies on event-based optical flow, faces the bottleneck of motion ambiguity. Since event data primarily captures intensity changes perpendicular to an object’s edges (normal direction) rather than the tangential direction, a single event stream can correspond to multiple valid motion vectors. This instability complicates the distinction between static backgrounds and independent moving objects, limiting the practical utility of event cameras.

In response to the aforementioned ambiguity in motion, this article first proposes a Motion Trend Map (MTM) data processing architecture. Discrete events are mapped into continuous 2D image representations to suppress the interference of motion ambiguity on segmentation and provide motion priors for the initial localization of independent moving objects while preserving the motion direction information of each target in the scene.

In addition, events are scarce inside the target or in low-contrast areas, resulting in incomplete scene structure and affecting segmentation accuracy. To this end, an event leaky integration (ELI) model is employed to enhance the edge features of scenes and moving targets, supplement missing structural details, and provide a more complete scene representation for segmentation.

A dual-branch segmentation network is built in this article. The Trend Branch utilizes MTMs for motion priors, while the Structure Branch processes ELI-enhanced representations for spatial context. Through multi-scale feature fusion, the network achieves high-precision segmentation of moving objects with minimal computational latency, effectively balancing accuracy and real-time performance.

## 2. Related Work

### 2.1. Frame-Based Motion Segmentation

Traditional motion segmentation primarily relied on frame-based visual sequences, where performance was inherently limited by fixed frame rates and exposure-related latency. Early approaches, such as frame differencing [7], detected motion via pixel-level temporal changes. However, these were highly sensitive to noise and struggled with multi-object scenarios. Edge-based methods relied on static texture assumptions, exhibiting poor robustness under weak textures or dynamic blur. To improve precision, optical flow was widely employed to estimate velocity fields by modeling spatio-temporal gradients [8,9]. Nevertheless, optical flow was fundamentally constrained by the brightness constancy assumption, which frequently failed in real-world conditions involving illumination changes, occlusions, or complex material properties. While subsequent research integrated camera poses, depth information, or dual-stream RGB-flow networks to enhance robustness, these solutions remained bottlenecked by the physical latency of frame-based data, failing to satisfy the requirements for real-time, high-precision mask segmentation in high-speed scenarios.

### 2.2. Learning-Based Methods for Conventional Vision

With the advancement of deep learning, Convolutional Neural Networks (CNNs) have become the mainstream for motion segmentation [10,11,12]. Architectures like U-Net utilize skip connections to fuse shallow and deep features, preserving critical spatial details. For instance, Tokmakov et al. extracted and fused feature maps from consecutive frames to generate motion masks, while Meunier et al. [13] explored self-supervised learning to reduce dependency on labeled data [14]. Despite these advancements, frame-based CNNs still face severe challenges [15]: high-speed movement causes significant motion blur and inter-frame temporal misalignment, while high dynamic range (HDR) scenes lead to information loss in over- or under-exposed regions and low signal-to-noise ratios [16].

### 2.3. Event-Based Motion Segmentation: Optimization Approaches

Event cameras offer a bio-inspired paradigm shift to overcome the aforementioned limitations. Event-based segmentation can be categorized into optimization-based and learning-based methods [17,18]. Optimization-based methods typically realize segmentation by minimizing cost functions or tuning hyperparameters [19,20]. Mitrokhin et al. [21] fitted a camera motion model using event timestamps to identify outliers as moving objects. Falanga et al. [22] further improved real-time performance and estimation accuracy by incorporating IMU sensors, while He et al. [23] refined timestamp representations by fusing depth information. Although these methods do not require labeled data, their computational efficiency is low, and their underlying assumptions of spatio-temporal coherence often fail in complex scenes due to conflicting motions or depth discontinuities.

### 2.4. Event-Based Motion Segmentation: Learning Approaches

Learning-based methods pre-process event data and feed it into architectures such as CNNs, Graph Neural Networks (GNNs), or Spiking Neural Networks (SNNs) [24]. The introduction of the EV-IMO dataset provided a high-quality benchmark with precise masks for evaluating these algorithms [25]. The original EV-IMO study proposed a U-Net-based scheme that accumulated event streams into three-channel slices; however, using five consecutive slices resulted in a 125 ms delay, limiting real-time utility.

Recent research tried to balance latency and accuracy. Mitrokhin et al. utilized Graph Neural Networks (GNNs), treating time as a third coordinate in 3D point clouds to extract motion trends, achieving a 52.4% mIoU with 30 ms event fragments. Parameshwara et al. introduced the first deep encoder-decoder SNN [24], which reduced power consumption through spatio-temporal loss formulations but achieved lower IoU than EV-IMO. Most recently, Shaobo et al. [26] reached a SOTA accuracy of 82% with a multi-scale Recurrent Neural Network (RNN). However, it still relied on 30 ms slices and incurred a total latency of 300 ms. While learning-based methods offered superior robustness in complex scenarios, their performance remained heavily dependent on designing network architectures that could resolve the trade-off between inference speed and segmentation precision.

## 3. Methodology

### 3.1. Overview of the Proposed Methodology

**Figure 1:**
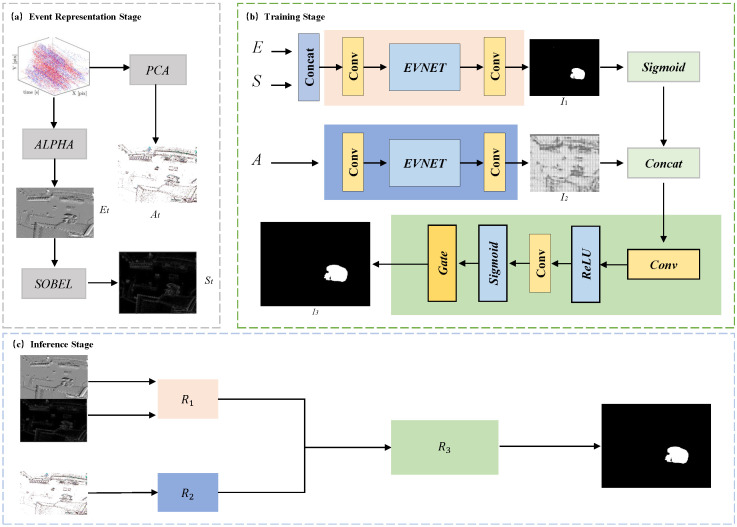
(**a**) The data preprocessing section explains how to generate MTM, ELI, and SOBEL graphs. (**b**) The proposed dual-branch network architecture: the main branch (Shape feature extraction branch) takes the event accumulation integral image and Sobel edge map as inputs, while the motion trend verification branch processes the motion trend map; the two branches respectively pass through four-level and three-level U-Net networks respectively, then feed into a spatial gating module for feature fusion, producing the motion segmentation mask. (**c**) The Inference Architecture of our proposed event-based network.

This paper presents a novel independent motion segmentation framework utilizing purely event-based data. To fully exploit the rich spatiotemporal information inherent in the asynchronous event stream, the proposed architecture consists of three core stages: event representation, training stage, and inference stage, as illustrated in Figure 1.

First, in the **Event Representation Stage**, the raw event stream is processed into distinct 2D representations to capture both motion trends and structural details. For motion representation, the event data is expanded into three dimensions along the time axis. Principal Component Analysis (PCA) is then applied, and the calculated values are mapped to the RGB color space based on their direction. By appending the event polarity as the final dimension (forming an RGBA format), this 3D data is projected into a 2D Motion Trend Map (MTM). Concurrently, for structural representation, an Event Leaky Integration (ELI) model is directly applied to the raw events to generate an ELI map. A Sobel operator is subsequently applied to this ELI map to extract refined and clearer scene structure information, producing a Sobel map.

Second, in the **Feature Extraction Stage**, these representations are fed into a customized dual-branch U-Net architecture (EVNET). The ELI map and the Sobel map are concatenated and inputted into the **structure branch** to capture high-resolution spatial contexts. Simultaneously, the MTM is inputted into the **motion branch** to learn complex kinematic patterns.

Finally, in the **Spatial Fusion Stage**, the extracted features from both the structure and motion branches are integrated through a **Spatial Fusion Gating Unit**. This unit dynamically weights and combines the multi-modal features, ultimately outputting an accurate, pixel-wise independent motion segmentation mask.

### 3.2. Event Data Representation and Preprocessing

The performance of current event-to-frame-based segmentation is hindered by two main bottlenecks: the absence of transient motion dynamics and the inherent sparsity of object geometry. This research tackles these limitations by implementing two critical refinements in the data representation pipeline.

#### 3.2.1. Motion Trend Mapping via PCA

Event data inherently comprise four-dimensional information. Conventional 2D representations, which project events solely via pixel coordinates, compress the temporal dimension and cause significant loss of dynamic cues. To preserve these cues, we represent events as 3D point clouds by utilizing timestamps as the third dimension. This representation facilitates the extraction of spatiotemporal correlations and geometric structures, thereby capturing critical motion information.

To manage the high computational load from millions of events per second, we perform dimensionality reduction on the event point cloud after temporal scaling. Given that individual event motion is difficult to track, we infer motion trends from local neighborhood distributions using Principal Component Analysis (PCA). Specifically, the event stream is first extended in the time domain. For each event, a spherical neighborhood with a 5-pixel radius is defined, within which PCA is applied to compute the characteristic matrix and the principal motion direction.

**Figure 2:**
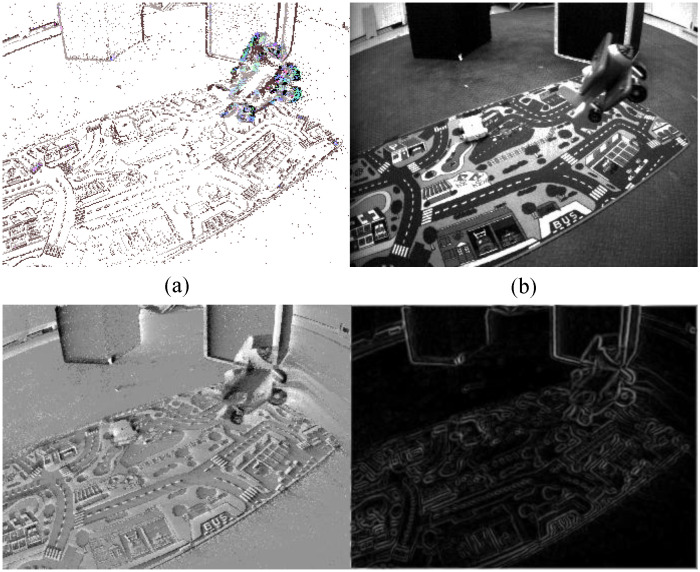
(**a**) Motion trend map, where the colors of the moving airplane are clearly distinguishable from the background motion; (**b**) Grayscale image of the current scene; (**c**) ELI map, which preserves substantial structural and textural details of the scene; (**d**) Sobel edge map derived from the ELI map, demonstrating its effectiveness in maintaining scene structural integrity.

The principal direction reflects the aggregate motion trend of local events. To utilize this information effectively, we map these 3D directions into the RGB color space, encoding 3D motion dynamics into a 2D color image (Figure 2a). This allows for an intuitive analysis of motion via color distribution, which exhibits rich variations in scenarios where both the camera and objects are in motion.

In the data preprocessing stage, to reduce the error of motion trend calculation, the time coordinate of the 3D event data is first scaled by a factor of 200. This specific scaling is crucial for spatio-temporal metric alignment, as it brings the temporal variance ($\sigma_{t}^{2}$) to a comparable order of magnitude with the spatial variance ($\sigma_{s}^{2}$), preventing the PCA from trivially aligning the primary eigenvector solely with the spatial plane. Subsequently, centered on each event point, the PCA main motion direction is calculated within a neighborhood with a spatial radius of 5 pixels. This radius is selected to fully encapsulate a single, continuous moving edge for a robust covariance matrix while strictly preserving the local planarity assumption required by PCA. Central event points with fewer than 30 events in this region are treated as isolated noise and ignored. This strategy effectively suppresses ambient thermal noise while reducing computational load, significantly improving the quality of the resulting motion trend map. The motion trend map is then generated according to the specified time slice.

#### 3.2.2. Event Leaky Integration Model

Furthermore, the non-uniform spatiotemporal distribution of event data—characterized by regions of high sparsity—makes it difficult to reconstruct scene structures within short time windows. To address this, we propose an event leaky integration model that transforms asynchronous event streams into grayscale images enriched with temporal information. By employing an exponential decay function, the model simulates the membrane potential dynamics of biological neurons. The update formulation is as follows:

(1) $$I\left(t\right)=I_{\mathrm{prev}}\times \exp\left(-\frac{t-t_{0}}{\tau }\right)+\sum_{i=1}^{n}C^{\prime }\cdot \left(2p_{i}-1\right)$$

where I(t) is the current pixel intensity, $I_{\mathrm{prev}}$ is the intensity at the previous time step, τ is the time decay constant, *n* is the cumulative event count for the current pixel, *C* is the event contribution coefficient, and p∈{0, 1} denotes the event polarity (positive/negative event).

Define the decay factor $\beta =\exp(-f_{\mathrm{cutoff}}\cdot \Delta t)$, where $f_{\mathrm{cutoff}}$ is the cutoff frequency (the reciprocal of the time decay constant), and Δt is the time difference. To prevent numerical overflow, taking the logarithm of the equation yields:

(2) $$\log I\left(t\right)=\beta \times \log I_{\mathrm{prev}}+\left(2p-1\right)\times C$$

For pixels that have not received event updates, exponential decay is applied, where α is used to control the global decay rate.

(3) $$\log I\left(x,y\right)=-\alpha +\log I^{\prime }$$

The leaky integration model operates via pixel-wise updates, aligning closely with the asynchronous triggering mechanism of event sensors. This approach enables the generation of event integral maps at any timestamp, thereby mitigating the information loss inherent in discrete event slicing. Consequently, the model effectively extracts structural details in complex dynamic scenes, providing accurate contour representations for subsequent independent motion segmentation.

For the event accumulation integral image, the parameters are set to α=0.27 and C=0.002, and the event update integral image is also generated. The parameter α is mathematically derived from the product of the cutoff frequency (30 Hz) and the global synchronization time interval (0.009 s). It acts as a unified global clock for the asynchronous event stream, applying a uniform global decay to inactive pixels to clear outdated motion histories (dead shadows) and ensure temporal consistency while avoiding massive computational overhead. The *C* value is set to 0.002 to act as a spatial low-pass filter. Because it is orders of magnitude smaller than the physical threshold, it prevents single, isolated background noise events from producing high responses, ensuring that only continuous event generation accumulates.

To provide a more intuitive understanding of the aforementioned data preprocessing and representation mechanisms, Figure 2 visualizes the intermediate outputs of our pipeline. Specifically, the figure consists of four distinct components: (a) the generated Motion Trend Map (MTM), which encodes the spatial-temporal kinematics into an RGB space; (b) the corresponding standard grayscale image for visual reference; (c) the Event Leaky Integration (ELI) map, which accumulates asynchronous events into a dense structural representation; and (d) the Sobel edge map, which extracts explicit spatial boundaries from the ELI map.

### 3.3. Dual-Branch Decoupled Segmentation Network

Event-based independent motion segmentation is a fundamental yet challenging task with broad application prospects in autonomous driving, video surveillance, and human-computer interaction. Unlike traditional frame-based segmentation, its primary difficulty lies in effectively leveraging continuous temporal information and high-resolution motion cues to handle complex scenarios, such as illumination variations, motion blur, and target occlusions.

#### 3.3.1. Structural and Motion Branches

As illustrated in Figure 2, the proposed research adopts a dual-branch network architecture designed to decouple the tasks of shape extraction and motion trend verification. This framework leverages the distinct characteristics of event integral maps and motion trend graphs, integrating their features through a spatial gating mechanism.

**Structural Feature Extraction Branch**: This branch takes the event leakage-integrate (LI) graph and its corresponding Sobel edge map as inputs, aiming to extract the contours and structural information of targets. The encoder-decoder is built upon a four-level U-Net structure, with the number of feature channels progressively expanding from 32 to 256. The decoder gradually restores spatial details via upsampling and skip connections with the encoder, ultimately producing the initial segmentation result (denoted as $I_{1}$).

**Motion trend verification branch**: The input is a motion trend map encoding event accumulation, direction, and motion information. This branch employs a three-level U-Net network to rapidly generate a single-channel motion confidence map $I_{2}$, providing region-level motion discrimination without focusing on fine-grained segmentation.

#### 3.3.2. Spatial Gated Fusion Module

The outputs from both branches are integrated through a key spatial gating fusion module. This module first normalizes the structural branch’s raw logits $I_{1}$ using the Sigmoid activation function σ to obtain probability map $P_{G}$. Subsequently, $P_{G}$ is concatenated with the motion confidence map $I_{2}$ along the channel dimension, and processed by a sub-network comprising convolutional layers and a Sigmoid activation function to generate the spatial gating map $G\in {\left[0,\;1\right]}^{H\times W}$. The final output $L_{F}$ is obtained by $P_{G}$ and *G*. The computation process is as follows:

(4) $$P_{G}=\sigma \left(I_{1}\right)$$

(5) $$G=\sigma \left(\mathrm{Conv}\left(\mathrm{Concat}\left(P_{G},I_{2}\right)\right)\right)$$

(6) $$L_{F}=P_{G}⊙G$$

This mechanism effectively suppresses the segmentation of static backgrounds from the shape branch, ensuring that the network retains only the independently moving objects. For network training, Binary Cross-Entropy (BCE) loss is employed, with the integration of label smoothing to mitigate overfitting:

(7) $$y^{\prime }=y\cdot \left(1-\epsilon \right)+\frac{\epsilon }{2}$$

(8) $$L_{\mathrm{BCE}}=-\left[y\log(p)+(1-y)\log(1-p)\right]$$

To further focus the model on hard-to-classify samples, we incorporated a moderate Focal Loss weighting strategy. Defining $p_{t}=\exp\left(-L_{\mathrm{BCE}}\right)$, the final optimization objective is:

(9) $$L_{\mathrm{opt}}=\frac{1}{N}\sum_{i=1}^{N}\alpha {\left(1-p_{t,i}\right)}^{\gamma }\cdot L_{\mathrm{BCE},i}$$

where the weighting factor γ is set to 1.0.

## 4. Experimental Results and Analysis

### 4.1. Dataset and Experimental Setup

The EV-IMO dataset contains approximately 30 min of data from diverse indoor scenes, providing precise ground-truth masks for moving objects at a frequency of 200 Hz. In this study, these masks serve as the ground truth for both model training and performance evaluation.

The network contains approximately 2 million trainable parameters and is trained using one NVIDIA GTX 3090 Ti GPU. The batch size is set to 8 (8 samples per network input). The Adam optimizer is employed with an initial learning rate of $3\times 10^{-4}$, which is dynamically adjusted.

### 4.2. Qualitative Results and Limitation Analysis

**Figure 3:**
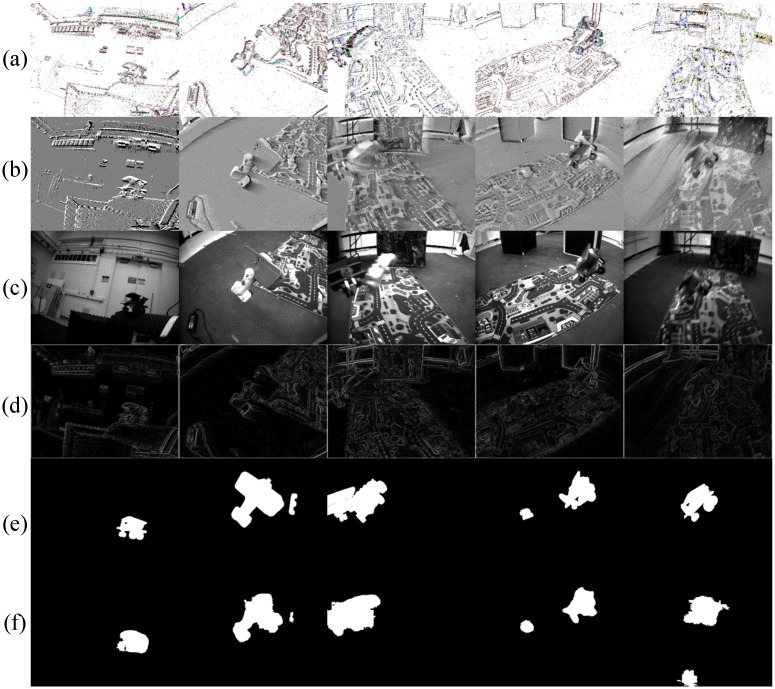
Qualitative segmentation results on the EV-IMO dataset. (**a**) Motion Trend Map (MTM); (**b**) Event Leaky Integration (ELI) map; (**c**) Grayscale images; (**d**) Sobel edge map; (**e**) Ground truth; (**f**) Proposed segmentation results.

To comprehensively evaluate the effectiveness of our proposed dual-branch U-Net architecture in dynamic scenes, we conducted a qualitative analysis on the EV-IMO dataset. As shown in Figure 3, we present the segmentation results across five different scenarios. To facilitate visual assessment, the figure includes not only the input Motion Trend Map (MTM, Row a) and Event Leaky Integration map (ELI, Row b), but also the corresponding grayscale images (Row c) and Sobel edge maps (Row d). These are compared against the ground truth labels (Row e) and our final segmentation results (Row f).

In typical motion scenarios (Columns 1, 2, and 4), the model demonstrates high accuracy and robustness. By effectively fusing the motion trend features from MTM and the local texture features from ELI, the model accurately identifies and segments independent moving objects. The predicted boundaries are clear and align closely with the ground truth.

Despite the overall robust performance, the model exhibits certain limitations and missegmentation issues under challenging conditions. Firstly, when multiple moving objects overlap or closely interact (as seen in Column 3), the model tends to merge them into a single dynamic region. The boundary textures between the overlapping objects are lost in the final segmentation mask. This occurs because our current architecture focuses on semantic-level motion segmentation rather than instance-level separation, lacking the mechanism to explicitly distinguish boundaries within a contiguous active event cluster.

Secondly, in scenarios with rapid camera or object motion (as seen in Column 5), the ELI representation suffers from severe motion blur or “ghosting” effects due to the accumulation of historical events over a fixed time window. This causes the extracted dynamic feature regions to become enlarged and blurry. Consequently, the model not only outputs a relatively blurred and oversized dynamic region but occasionally misclassifies adjacent static background edges as dynamic objects, resulting in false positives.

To address these limitations in future work, we plan to focus on three main avenues. First, we will explore more advanced event representation methods to fundamentally resolve the ghosting effects inherent in the current ELI approach, aiming for sharper structural extraction. Second, we will investigate more precise motion differentiation algorithms to refine the PCA-based motion trend calculation, reducing misjudgments in complex kinematics. Lastly, developing more adaptable network architectures and stronger backbones tailored for event streams will be crucial for improving the model’s robustness in highly dynamic and unconstrained environments.

### 4.3. Experimental Results

Table 1 presents the performance comparison of various motion segmentation methods on the EV-IMO dataset. To facilitate a clear comparison of the trade-off between temporal latency and accuracy, the specific event window size utilized by each method is explicitly listed.

Additionally, we compared our method with other motion segmentation approaches based on the EV-IMO dataset, with results presented in Table 1. Considering that one of the key advantages of event cameras over traditional frame-based cameras is their high temporal resolution (up to 1 μs), which provides inherent advantages in applications such as autonomous driving and human-computer interaction, event data processing should ideally utilize narrower time window event slices for lower temporal latency. Following the development in this field, our method employs an ultra-short 0.01 s event slice for inference.

When observing the results, it is essential to objectively discuss the trade-off between temporal latency and segmentation accuracy. Methods utilizing longer time windows inherently accumulate more events, providing denser structural and texture information. This is particularly advantageous for segmenting stable, continuous background elements or scenes with slower dynamics. For instance, the MSRNN method, which uses a 0.03 s event slice and temporarily stores structural information via a multi-scale recurrent neural network, achieves higher accuracy than our method in certain structurally rich scenarios, such as the floor (85% vs. 81%), wall (82% vs. 73%), and table (85% vs. 80%) scenes. Similarly, methods like GCN can achieve competitive accuracy by utilizing even longer observation windows (e.g., 0.125 s) to build comprehensive spatial graphs.

However, the reliance on longer observation windows introduces higher latency, which can be a critical bottleneck in time-sensitive applications. Therefore, the applicability boundary of our proposed method lies in highly dynamic, ultra-low latency scenarios where rapid system reaction is paramount. By utilizing a 0.01 s window, our approach intentionally trades a marginal decrease in accuracy in certain structured scenes for a substantial reduction in temporal latency compared to MSRNN and GCN. Notably, even with this heavily constrained time window, our method significantly outperforms GCN and GCN2’s 0.03 s results across all scenarios and maintains competitive performance in the fast dynamic scene.

In conclusion, longer-window methods (like MSRNN or GCN with 0.125 s slices) should be preferred when structural completeness and maximum segmentation accuracy are the primary goals. Conversely, our method should be prioritized when system responsiveness and minimizing latency are the strict limiting factors.

### 4.4. Ablation Studies

As shown in Table 2, the IoU results of the Event Leaky Integration (ELI) map (Single-Branch ELI) significantly outperform the standard event accumulation map (Single-Branch Accum) across all scenes, particularly with substantial margins in the wall and fast scenes. Compared to conventional accumulation that suffers from severe temporal decay and information loss in rapid movements (e.g., the fast sequence), the ELI map employs an exponential decay function. This temporal continuity effectively preserves the structural topology of moving objects, making it a robust foundational feature.

To directly address the necessity of the dual-branch architecture, we explicitly decouple and evaluate the individual contributions of the structural and motion representations. While the Single-Branch ELI network achieves a commendable baseline by effectively extracting spatial features, it inherently lacks kinematic awareness. Consequently, it struggles to differentiate between true independently moving objects and static background edges triggered by the camera’s ego-motion, leading to noticeable false positives. Conversely, the Single-Branch MTM network, which relies solely on motion priors, experiences a catastrophic performance drop to below 5% across all scenarios. This failure highlights that while the MTM captures critical motion trends, it lacks the dense spatial resolution required to delineate precise object boundaries. Therefore, a dual-branch design is structurally imperative: the ELI branch provides the indispensable high-resolution spatial boundaries (the structural anchor), while the MTM branch contributes the highly concentrated kinematic prior necessary to resolve dynamic ambiguities.

Building upon this complementary design, we ablate the fusion strategy. Although feature concatenation (Dual-Branch Concat) improves upon the single-branch baselines by forcing the network to learn from both modalities, it often suffers from feature interference and suboptimal noise suppression. In contrast, our proposed Spatial Gating mechanism mathematically aligns with the dual-branch philosophy: it utilizes the highly discriminative motion prior from the MTM branch as an active spatial filter to selectively gate the structural features from the ELI branch. This targeted cross-modal interaction effectively masks out ego-motion-induced static noise, enabling our model to achieve the highest IoU across all scenarios and rigorously proving the efficacy of our decoupled feature extraction and fusion strategy.

### 4.5. Comprehensive Multi-Metric Evaluation

To provide a more holistic understanding of the model’s performance beyond traditional region-overlap metrics (IoU), we conducted a comprehensive evaluation using a broader set of indicators: Precision, Recall, F1-score, Boundary F-score, and Temporal Consistency (TC). Because event cameras inherently capture high-frequency motion edges and possess high temporal resolution, these supplementary metrics are essential for assessing the structural sharpness and temporal stability of the predicted segmentation masks. The detailed results across five diverse scenarios in the EV-IMO dataset are presented in Table 3.

As indicated in Table 3, the proposed model demonstrates a stable region-level tracking capability in standard scenarios (boxes, floor, and table), achieving F1-scores between 77.63% and 78.32%. The model provides a reasonable balance between Precision and Recall. However, in the more challenging wall scenario, overall performance observes a moderate decline, indicating that scenes with ambiguous motion boundaries or specific textures still pose a challenge for pure event-based segmentation.

A distinct theoretical advantage of event data is its sensitivity to object boundaries. To objectively quantify this, we evaluated the Boundary F-score. Unlike region-based F1-scores, pixel-level boundary matching is exceptionally strict. Therefore, we adopted a physically realistic distance tolerance of ϵ=2 pixels. This specific tolerance accounts for the spatial jitter of event triggering, the motion smear within the 0.01-second accumulation slices, and the modality alignment gap between asynchronous events and synchronous ground-truth frames. The model achieves boundary scores ranging from 32.39% to 46.07% in standard scenes. Considering the inherent spatial noise and defocus effects of event cameras, these results establish a solid and realistic baseline, demonstrating that the integration of motion trend maps helps preserve the sharp contours of moving objects without the severe motion blur typical of frame-based sensors.

Furthermore, the Temporal Consistency (TC) metric validates the temporal smoothing effect of the Event Leaky Integration (ELI) module. In regular scenarios, the TC ranges from 52.08% to 78.16%, reflecting a reduction in prediction flickering across consecutive frames. As physically expected, the TC experiences a significant drop to 36.59% in the fast scenario. This decrease is primarily attributed to the drastic inter-frame spatial displacement caused by high-speed motion, which naturally reduces the spatial intersection ($Pred_{t}\cap Pred_{t-1}$) between consecutive predictions. Despite this limitation in temporal overlap, the model still maintains a functional tracking performance (F1-score of 61.04%) under extreme dynamic conditions.

### 4.6. Ablation Study on Hyperparameters

To rigorously validate our parameter settings and investigate their sensitivity, we conducted a comprehensive fine-grained ablation study on the Focal Loss parameter, γ, extending the analysis to γ∈{1.0, 1.2, 1.5, 2.0, 3.0}. In conventional RGB-based object detection tasks, Focal Loss is typically configured with γ≥2.0 to force the network to concentrate on hard-to-classify, occluded examples. However, the nature of pure event data presents a fundamentally different paradigm.

**Figure 4:**
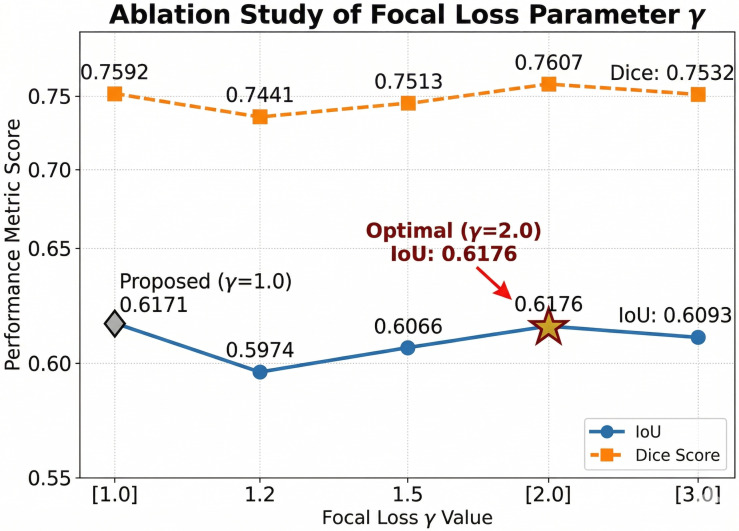
Ablation study of the Focal Loss parameter γ evaluated on the full dataset, extending to γ=3.0. The proposed setting (γ=1.0) and the standard optimal setting (γ=2.0) yield nearly identical optimal performance. Crucially, a rapid performance drop occurs at γ=3.0, demonstrating that a lower γ is sufficient and necessary to prevent over-fitting inherent hardware noise in pure event data.

As illustrated in Figure 4, we evaluated the segmentation performance across five different γ values using the full dataset. The results indicate that the performance initially drops as γ increases from 1.0, before recovering at γ=2.0. While the empirical setting (γ=2.0) achieved the peak IoU of 0.6176, it only outperformed our proposed setting (γ=1.0, IoU: 0.6171) by a statistically negligible 0.05%.

This phenomenon can be attributed to the inherent characteristics of event cameras. Unlike standard images, the background in pure event data is intrinsically unstructured and contains considerable sensor noise and isolated event spikes. From the perspective of the loss function, these random noise points act as extreme “hard negative examples.” Setting γ too high (e.g., ≥3.0) aggressively penalizes the network for these unavoidable hardware noises. As shown in Figure 4, this design choice theoretically and empirically leads to a rapid degradation in segmentation performance, as the IoU falls significantly to 0.6093 at γ=3.0, demonstrating an over-focusing on background artifacts rather than true dynamic objects.

Therefore, we deduce that γ=1.0 provides the optimal balance. It acts as a deliberate and smoothly weighted Binary Cross-Entropy (BCE) that successfully suppresses dominant event-free background areas while preventing the over-amplification of noise gradients, ensuring a robust and reliable independent motion segmentation suitable for subsequent dynamic SLAM feature tracking.

### 4.7. Computational Complexity and Efficiency

To evaluate the feasibility of deploying the proposed decoupled MTM and ELI framework on embedded systems, we thoroughly analyzed its computational complexity and memory footprint during inference. Given an input resolution of 352×320, the overall network requires 18.09 GFLOPs and contains 1.94 M trainable parameters. Crucially, our dual-branch architecture employs a highly efficient asymmetric design. While the structural branch processes dense spatial contexts, the proposed lightweight motion verifier—the core module responsible for processing the MTM features—extracts highly concentrated kinematic priors. This verifier accounts for merely 59.70 M FLOPs (≈0.33% of the total computation) and 8.88 K parameters (≈0.45% of the total footprint). This demonstrates that the MTM branch effectively resolves dynamic object ambiguities by providing a highly cost-effective motion prior, significantly enhancing segmentation accuracy with almost negligible computational overhead.

Regarding physical power consumption, the current experiments on the EV-IMO dataset were conducted on a desktop GPU. While accurate hardware-level power profiling requires specific embedded edge platforms, our highly compact 2D convolutional architecture is inherently optimized for modern hardware accelerators and allows for seamless integration with matrix-based visual SLAM systems. Deploying this lightweight module onto embedded platforms to conduct real-time power evaluation remains a primary focus of our future work.

## 5. Discussion and Limitations

While the proposed MTM+ELI framework demonstrates a competitive balance between segmentation accuracy and inference efficiency in low-latency scenarios, several constraints define its current operational boundaries.

First, the framework is specifically designed for indoor robotic applications. The PCA-based Motion Trend Map (MTM) is highly effective at capturing local motion trends of rigid or piecewise-rigid objects within structured environments. However, in unconstrained outdoor settings, intense solar illumination can introduce substantial Background Activity (BA) noise and pixel saturation. Such non-ideal event distributions may pose challenges for the MTM in distinguishing subtle motion vectors from heavy noise backgrounds.

Second, our current approach assumes a certain level of structural rigidity in the moving targets. For complex non-rigid deformations—such as moving pedestrians with flowing clothing—a single linear motion trend might not capture the full complexity of the underlying physics. Future research will focus on integrating adaptive filtering techniques and more flexible event representations to further enhance the robustness of this trade-off against extreme lighting conditions and non-rigid motions.

Third, and most importantly regarding the broader scope of this work, the highly accurate independent motion masks generated by our framework hold significant potential for downstream visual Simultaneous Localization and Mapping (SLAM) applications. In dynamic environments, moving objects inevitably introduce erroneous feature associations, which severely degrade camera tracking and cause substantial pose estimation drift. Our future work will focus on integrating this event-based segmentation model as a robust front-end filter for visual SLAM systems. By reliably identifying and rejecting dynamic feature points prior to pose optimization, our methodology aims to effectively mitigate tracking drift and substantially improve the overall accuracy and robustness of SLAM algorithms in complex, dynamic scenes.

## 6. Conclusions

Addressing the challenges of motion ambiguity and data sparsity in event-based independent motion segmentation within indoor dynamic environments, this study proposes a framework focused on efficient data representation and decoupled learning. By introducing Motion Trend Maps (MTM), we model and preserve the local spatiotemporal motion priors of events, thereby mitigating uncertainty in motion estimation for structured scenes. Simultaneously, the Event Leaky Integration (ELI) model enhances the structural details of sparse events, providing a more robust representation for the segmentation task. Building upon these representations, the developed dual-branch gated fusion network successfully decouples motion verification from shape extraction, achieving a favorable balance between segmentation precision and computational efficiency.

Experiments on the EV-IMO dataset demonstrate that with an event slice of only 10 ms, the proposed method not only outperforms the baseline using simple event accumulation but also maintains competitive performance compared to existing methods that rely on longer event windows. This validates the framework’s capability to effectively parse scene motion within ultra-short time windows, offering a practical technical pathway for latency-sensitive indoor applications, such as service robotics and warehouse automation. While further research is needed to extend this robustness to unconstrained outdoor environments and non-rigid motions, the current work provides a solid foundation for real-time event-based perception.

## Figures and Tables

**Table 1:** mIoU scores comparison across different methods and time windows.

Method	Window (s)	Boxes	Floor	Wall	Table	Fast
Ours	0.01	82	81	73	80	70
MSRNN	0.03	79	85	82	85	76
GCN	0.125	81	79	83	80	74
	0.03	60	55	51	57	39
GCN2	0.125	70	69	71	77	69
	0.03	54	52	61	59	37
Pointnet++	0.125	71	68	75	62	24
	0.03	80	76	74	68	20
EV-IMO	0.125	70	59	78	79	67

**Table 2:** Ablation study results: Comparison of IoU accuracy (%) across five scenarios.

Model Variants	Boxes	Floor	Wall	Table	Fast
Single-Branch (Accum)	47.65	49.87	21.45	46.51	28.36
Single-Branch (ELI)	55.01	52.89	46.27	51.34	42.57
Single-Branch (MTM)	<5.00	<5.00	<5.00	<5.00	<5.00
Dual-Branch (Concat)	58.41	61.69	46.56	60.23	43.03
Proposed (Ours)	64.37	63.48	50.78	63.44	43.93

Note: Single-Branch (Accum) uses standard event accumulation maps; Single-Branch (ELI) uses Event Leaky Integration maps; Single-Branch (MTM) uses only Motion Trend Maps; Dual-Branch (Concat) utilizes simple feature concatenation; Proposed uses our Spatial Gating fusion strategy.

**Table 3:** Comprehensive evaluation of the proposed method across multiple metrics and scenarios. All values are reported as percentages (%).

Metric	Boxes	Floor	Wall	Table	Fast
IoU	64.37	63.48	50.78	63.44	43.93
Precision	75.04	78.72	68.14	86.82	59.00
Recall	81.90	76.62	66.59	70.19	63.22
F1-score	78.32	77.66	67.36	77.63	61.04
Boundary F-score	42.03	46.07	32.39	36.22	23.42
TC	78.16	69.90	52.08	72.32	36.59

Note: Precision, Recall, and F1-score evaluate the region-level segmentation accuracy. Boundary F-score measures the structural contour alignment accuracy within a relaxed pixel tolerance. Temporal Consistency (TC) quantifies the prediction stability and smoothness across consecutive frames.

## Data Availability

The original data presented in the study are openly available in the EV-IMO Repository, reference number [25]. The processed data and experimental results generated during the study are available from the corresponding author upon request.
